# Tuning Carbon Dots’ Optoelectronic Properties with Polymers

**DOI:** 10.3390/polym10121312

**Published:** 2018-11-27

**Authors:** Konstantinos Dimos

**Affiliations:** Department of Materials Science & Engineering, University of Ioannina, GR-45110 Ioannina, Greece; kdimos@cc.uoi.gr; Tel.: +30-265-100-7367

**Keywords:** carbon dots, polymers, surface passivation, functionalization, photoluminescence, optoelectronic properties

## Abstract

Due to their unique properties of photoluminescence, biocompatibility, photostability, ease of preparing, and low cost, carbon dots have been studied extensively over the last decade. Soon after their discovery, it was realized that their main optical attributes may be protected, enhanced, and tuned upon proper surface passivation or functionalization. Therefore, up to date, numerous polymers have been used for these purposes, resulting to higher-quality carbon dots regarding their quantum yield or further emission-related aspects and compared to the primitive, bare ones. Hence, this review aims to clarify the polymers’ role and effect on carbon dots and their features focusing on the quality characteristics of their photoluminescence upon passivation or functionalization. Given in fact the numbers of relevant publications, emphasis is given on recent articles capturing the latest advances for polymers in carbon dots for expanding emission lifetimes, advancing quantum yields, tuning emission wavelengths, enhancing specific spectral range absorption, and tailoring optoelectronic properties in general.

## 1. Introduction

The topic of carbon dots has emerged during the last decade as one of the most promising fields of materials research, as revealed by the growing number of relevant publications in the literature. This is a clear indication for non-experts on the field and scientists exploring different topics that a potential breakthrough may lay under this burst of research activity. However, why do more and more scientists and research groups deal with carbon dots recently?

The answer is for their two combined and most significant features, i.e., photoluminescence and biocompatibility. Carbon dots possess analogous optoelectronic properties to conventional inorganic semiconductors [1,2]. Nevertheless, at the same time carbon dots not only are not toxic but in fact are biocompatible and biodegradable. On the other hand, organic fluorophores may be endowed with high luminescence and wide spectral range but lack photostability. However, it has been demonstrated lately that carbon dots may also provide high quantum yields with reported values up to 94% [3,4,5] added to high stability, being very resistant to photo-bleaching in contrast to commercially available photoluminescent dyes [6]. Furthermore, they probably represent the easiest to prepare and lowest-cost nanomaterial of all (unless found in nature). Since preparation is as simple as heating up for a few minutes in a domestic microwave oven a mixture of lemon juice (citric acid) and urine (urea), during past years numerous possible carbon waste materials have been successfully used for obtaining carbon dots [2]. The outcome is a truly valuable final nanomaterial in terms of extended applicability on diverse fields of research and technology [7] with bio-applications being on the spotlight [8,9,10,11,12,13]. Thus, carbon dots gained their recognition and space in materials research owned to the multiple advantages they offer, while their shortcomings compared to their competitors, organic dyes and inorganic semiconductors, namely quantum yield and spectral range, are being addressed effectively.

Since that time, researchers have been trying to produce higher quality carbon dots with respect to quantum yields, stability, narrow or tuned emission wavelengths, etc. Here is the point where polymers, among further agents, take part in advancing properties. It was not long after their accidental discovery as by-products [14] when it was realized that polymer passivation of carbon dots significantly enhances their fluorescence [15]. Ever since, various polymers have been used as pristine materials, passivating or functionalization agents leading to the formation of higher quality carbon dots [16].

Given the number of research groups working on carbon dots lately, even if focusing only on polymers’ effect, there is a vast number of relatively new publications on the topic, which is impossible to follow, let alone refer and cite. Hence, this review emphasizes a carefully selected small portion of recently published articles that demonstrate critical input on optoelectronic properties of carbon dots aiming to capture the latest advances on the field with regard to polymer usage.

## 2. Enhancing Carbon Dots’ Features Via Passivation

Carbon dots are being studied extensively with much effort given on unveiling the origin of their optoelectronic properties [17]. Although this is a point that will probably never clear up 100% due to the complex and undefined structure of carbon dots, it is now commonly accepted that the two main absorption bands originate from π–π* and n–π* transitions [18]. Since, in addition, surface groups and traps possess a significant role in the emissive process, it becomes clear why polymer passivation protects and enhances the fluorescence features of carbon dots leading in fact to much higher quantum yield values and thus fluorescence intensity. Furthermore, passivation radically improves carbon dots’ stability as their highly reactive and vulnerable surface groups are shielded beneath the polymer layer.

Various polymers have been used for this reason with poly(ethylene glycol) (PEG) being by far the most commonly employed one. Other frequently used polymers include polyethyleneimine (PEI) and block copolymers of the aforementioned PEG and PEI, as well as further nitrogen- and oxygen- bearing polymers as poly(vinyl alcohol) (PVA), etc. Table 1 lists the polymers used in the recent studies that are cited and discussed in detail on this review article further below.

## 3. Tuning Optical Properties with Polymers

When it comes to advancing of carbon dots’ properties, rationally the attention is focused on the optical ones. Therefore, in the following subsections, it is discussed how polymers can affect and improve various characteristics as the emission lifetimes, the quantum yields, the emission wavelengths, etc.

As already mentioned, the photoluminescence mechanisms of carbon dots are not fully unveiled. Consequently, the polymers’ effect on their optoelectronic properties, even if observed, may also be unclear or only partly explained to a certain point. According to recent experimental and theoretical studies on the photoluminescence origin of carbon dots [17,31,42,43,44,45,46,47,48,49], there are quite a few proposed mechanisms including quantum size effect and conjugated π-domains, surface states, molecule states, and crosslink-enhanced emission (CEE) effect, all of which are very well described in the work of Zhu et al. [50]. More (surface states and CEE) or less (quantum size effect, molecule states), polymers used for either synthesis, passivation, or functionalization of carbon dots are expected to influence all possible photoluminescence mechanisms. Nonetheless, the complexity of photoluminescence mechanisms and the variety of final carbon dots in terms of demonstrated optoelectronic properties and regardless of employing same precursors, polymers, or methodologies, imply that, concerning carbon dots’ optoelectronic properties, on top of all there is probably an unavoidable randomness since carbon dots’ synthesis is not a simple A plus B giving C chemical reaction. This means that even if the photoluminescence mechanisms of carbon dots are fully understood in the future, a step further for desired tuning will definitely be made, but still, due to their complexity, a complete predetermination of their properties will not be efficient.

### 3.1. Expanding Emission Lifetimes

**Figure 1:**
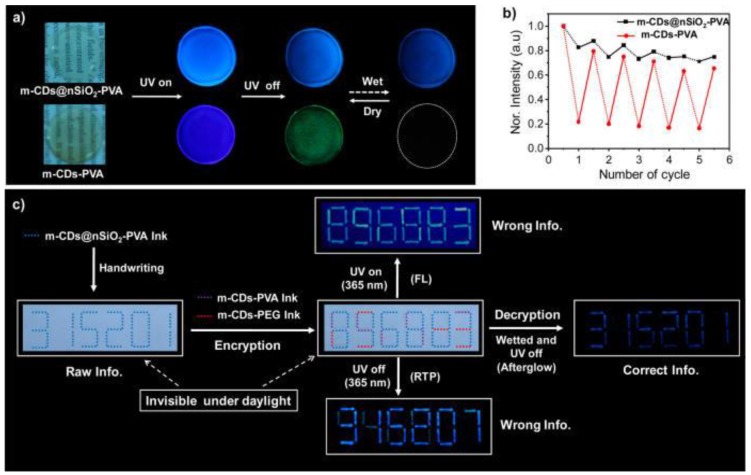
Expanding emission lifetimes of carbon dots by forming hydrogen bonds with PVA that restrain triplet emission species leading to room temperature phosphorescence [19,20]. Information protection applications of m-CDs@nSiO_2_. (**a**) Photographs of m-CDs@nSiO_2_-PVA and m-CDs-PVA films under daylight, and irradiation with 365 nm UV lamp on and off when the films are desiccated and wetted; (**b**) Intensities of afterglow alterations of m-CDs@nSiO_2_-PVA and RTP changes of m-CDs-PVA films under dry and wet conditions for five cycles; (**c**) Schematic illustration of information protection (encryption and decryption) processes by using m-CDs@nSiO_2_-PVA, m-CDs-PVA, and m-CDs-PEG inks. (Reproduced with permission from ref. [19]. Copyright 2017 American Chemical Society, Washington, DC, USA).

One very promising field on carbon dots research is for sure the expansion of their emission lifetimes so as to create afterglow materials. Whereas overwhelming majority (close to absolute) of carbon dots literature is connected to typical fluorescence, there are a few recent reports of carbon-dots-based materials exhibiting delayed fluorescence, persistent luminescence, or even phosphorescence [19,20,21,51,52,53,54]. An easy approach to achieve room temperature phosphorescence (RTP) is suggested by Professor Lin’s group [19,20]. In their experiments, PVA is used as a hydrogen bonding agent that stabilizes surface moieties that are responsible for triplet emission restraining their intramolecular motions. Hence, lifetimes of the carbon dots emissions expand from a few ns to several hundred ms. Final materials can be used in light-emitting devices, sensing, biomedicine, and security systems for anti-counterfeiting and information encryption (Figure 1). Encapsulation of carbon dots in polyurethane (PU) matrix may give rise to room temperature delayed fluorescence and phosphorescence of a few ms as well [21]. Again, the polymer matrix plays a key role in suppressing the nonradiative transitions of triplet excitons by rigidifying surface groups, thus enabling phosphorescence.

### 3.2. Advancing Quantum Yields

It is a very common fact in science and technology to have efficiency limitations on specific conversions. For sure, conversion of incident light to induced luminescence cannot be compared to more complex energy conversions; nevertheless, reaching a quantum yield of 94% [3] for carbon dots with passivation is a real breakthrough for their effective employment on widespread applications. This has been achieved by using *N*-isopropylacrylamide as a monomer passivating agent adopting the “monomer as solvent” approach [21,55] as it forms an amorphous layer around carbon dots core. A facile in situ hydrothermal synthesis is followed with *N*-isopropylacrylamide, not only contributing to the carbon dots core development together with citric acid and ethylenediamine but also creating the passivating matrix at the same time. This action gives rise to as monodisperse as possible carbon dots, preventing aggregation as well. Thus, the quantum yield of 94%, which is the second highest reported value until now for carbon dots—just 0.5% below the one in Reference [4]—may be the result of synergetic effect by the multiple roles of the *N*-isopropylacrylamide monomer during the synthesis, i.e., acting effectively for passivation, stabilization, size control, and agglomeration prevention [3].

**Figure 2:**
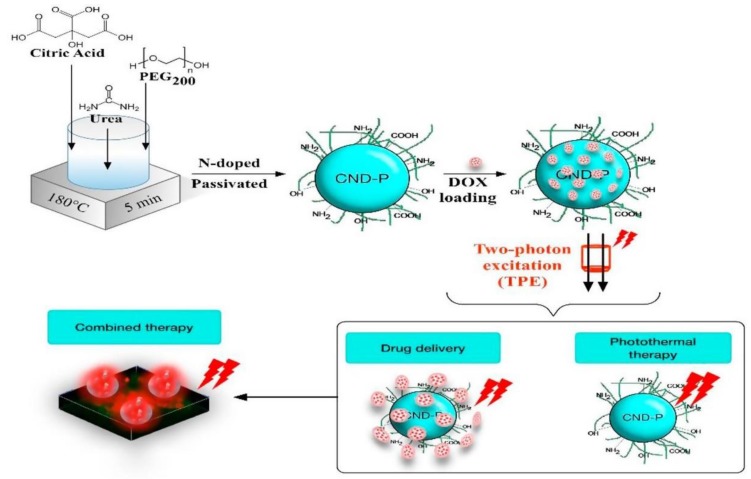
Schematic diagram of the synthesis procedure of biocompatible PEG-passivated carbon dots with a quantum yield of 53% for multifunctional on-demand drug release and combined chemo-photothermal therapy via two-photon excitation. (Reproduced with permission from ref. [22]. Copyright 2017 Elsevier B.V., Amsterdam, Netherlands).

Another recent example of the beneficial process of polymer passivation of carbon dots is the work of Ardekani and colleagues [22]. A one-pot synthetic procedure was followed where a mixture of citric acid, urea, and PEG was heat-treated at 180 °C while materials without PEG, or only by citric acid were also prepared with the same method for comparison reasons. As expected, the passivated carbon dots exhibited improved quantum yield reaching 53%, whereas the carbon dots without PEG had a 14% quantum yield and the ones originated only by citric acid had a quantum yield of as low as 4%. In fact, the improvement of quantum yield by a factor of ~4 or even more than 10, signifies the value of passivation but is not the only advancement since for instance the stability of the dots is also improved. Exploiting the high quantum yield, the PEGylated carbon dots were utilized for multifunctional on-demand drug release and combined chemo-photothermal therapy via two-photon excitation (Figure 2). This was based on an additional attribute of the passivated carbon dots which is their ability to bound molecules, as doxorubicin in this specific case, by weak interactions as forming hydrogen bonds with the passivating layer.

An alternative approach for enhanced photoluminescence is described by Momper and co-workers [27]. Whereas in typical carbon dots, polymers are used to form a protective cell, in Reference [27] carbon dots are created in situ or attached by mixing onto polystyrene (PS) nanoparticles. In fact, as assumed by experimental data, in situ formation leads to covalent attachment of the carbon dots on the PS nanoparticles and mixing provides composites by physical interactions, i.e., electrostatic attraction of the dots by the negatively charged PS nanoparticles. Thus, final carbon dots demonstrate a strong, ~5-fold enhancement in fluorescence intensity for both fabrication methods compared to the same unbound carbon dots, while the emission maximum is not shifted and a small extension by a few ns of their emission lifetimes is observed. Nevertheless, it should be noted here that only a relative enhancement of the luminescence is mentioned, and no actual quantum yield measurements are presented which means that regardless the enhancement, final quantum yields may still be low. On the other hand, though, this method is a truly alternative approach for enhancing fluorescence intensity that, if further exploited, may lead to high quantum yield values.

### 3.3. Tuning Absorption/Emission Wavelengths

Given the origin of carbon dots’ luminescence, i.e., π–π* and n–π* transitions [18] which lay on UV-C/UV-B and UV-A regions respectively, it is rational why most works on carbon dots report emissions at the closest to UV region of the visible spectrum (blue). However, and since carbon dots are extremely versatile materials, it is very important to be able to tailor their emission to further (longer) wavelengths so as to span across the visible spectrum and why not cover the near-infrared (NIR) region as well, which is known to be essential for biology and medicine. This is because NIR radiation is less scattered and absorbed by biological tissues compared to visible light thus can penetrate tissues such as skin and blood at a depth of 1 to 2 cm. Hence, the region from 650 to 950 nm is regarded as an optical window (NIR-I window) for in vivo imaging. However, carbon dots, being highly luminescent, biocompatible, and versatile, could be tuned to exploit a second biological window (NIR-II window) at even longer wavelengths (1000 to 1350 nm) which is considered to have improved penetration depths and signal-to-noise ratios, allowing deeper and more sensitive in vivo imaging [56]. Therefore, recent reports on both visible and infrared regions are discussed in the following subsections, as the visible range is important for optoelectronic applications and the NIR for bioimaging and photothermal therapy [25,26,33,34,35,36,38,39,44,49,57,58,59,60,61,62,63,64,65,66,67,68,69].

Besides this, Bao et al. demonstrated that by either changing the surface chemistry or increasing the size of carbon dots, their luminescence may be tuned to longer wavelengths [48], a behaviour observed by others as well [44,70]. This is because the photoluminescence is a function of the surface-state electronic transitions. Thus, energy gap of the surface states is narrowing upon increasing the degree of surface oxidation or the size of the carbon dots, since the larger the carbon dots, the more extensive the π-electron system which can couple with surface electronic states adding energy states. Therefore, polymers are ideal candidates for tailoring the luminescence at longer wavelengths.

#### 3.3.1. Visible Spectrum

**Figure 3:**
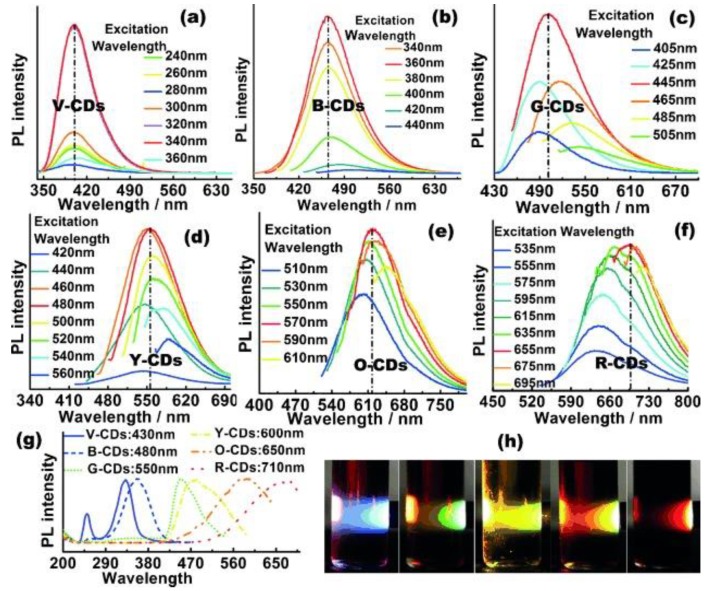
Tuning carbon dots’ emission to violet, blue, green, yellow, orange, and red: (**a**–**f**) PL spectra of V-, B-, G-, Y-, O-, and R-CDs, showing different strongest emission peaks; (**g**) PLE spectra of the six samples; (**h**) emission photos of different carbon dots excited by white light. (Reproduced with permission from ref. [31]. Copyright 2015 John Wiley & Sons, Hoboken, NJ, USA).

Since the visible spectrum is next to UV and spans from ~400 nm to ~700 nm, covering a small portion of the electromagnetic radiation, it is logical that the photoluminescence of carbon dots can be tuned to cover the visible region. In this direction, Hu et al. synthesized carbon dots with tailored photoluminescence across the entire visible spectrum when excited by white light [31]. Ethylenediamine end-capped PEI was used for the preparation of the carbon dots by mixing with either citric acid or ethylene glycol as carbon source. In both cases, carbon dots with main PL emission peaks at 530–550 nm were obtained. Nevertheless, when a reducing agent, i.e., NaBH_4_, was added in the starting reaction system of citric acid, the formed carbon dots exhibited blue-shifted PL emissions from violet to yellow, depended on the amount of NaBH_4_ added. In contrast, when a dehydrating agent, i.e., H_3_PO_4_, was added in the ethylene glycol system, the PL emissions were red-shifted from yellow to red. Hence, the final carbon dots displayed an excitation-independent PL emission, which was directly correlated to the oxygen and nitrogen containing groups of the carbon dots and covered the entire visible spectrum displaying violet, blue, green, yellow, orange, or red photoluminescence (Figure 3).

**Figure 4:**
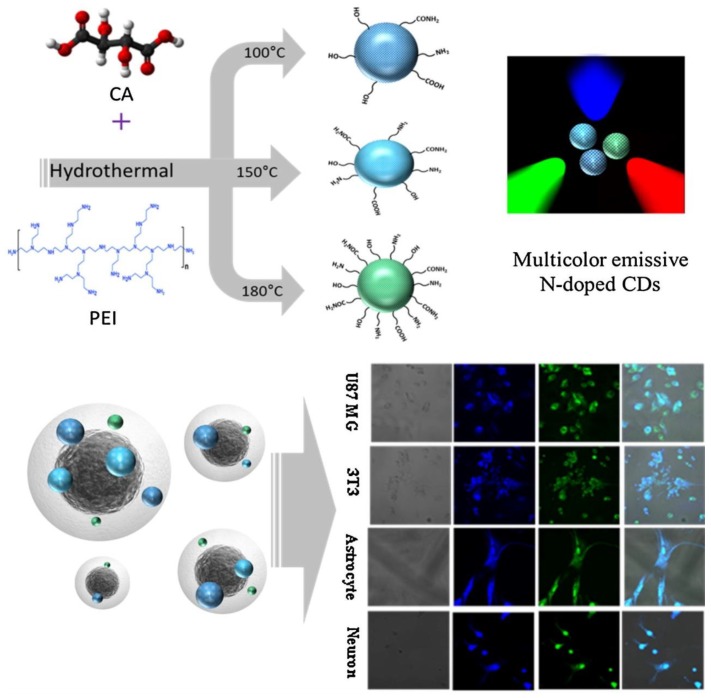
Schematic illustration for the synthesis of surface functionalized carbon dots with PEI and its application for in vitro cellular imaging. (Reproduced with permission from Reference [32]. Copyright 2018 Elsevier B.V.).

PEI was also used by Kundu and colleagues to obtain multicolour emissive nitrogen-doped carbon dots [32]. In their approach, b-PEI was mixed once again with citric acid and a hydrothermal reaction at variable temperatures (100 to 180 °C) and reaction times (5 to 20 h) took place. As a result, carbon dots with relatively high quantum yield values ranging from 33.7% to 56.2% were retrieved and were successfully employed for human brain tumour, fibroblast, neuron, and astrocyte cells imaging (Figure 4). It was also deduced that the carbon dots synthesized at higher temperature (180 °C) possessed improved fluorescence properties due to better surface functionalization and N-doping by b-PEI. Nonetheless, it should be noted here, in order to highlight also the significance of the previous described work by Hu et al. [31], that the multicolour emission in Reference [32] is based on the use of appropriate excitation wavelength (*λ*_exc._ = 405 nm for blue, 488 nm for green, and 555 nm for red emission), whereas in contrast in Reference [31] all carbon dots materials are excited by the same white light [32].

**Figure 5:**
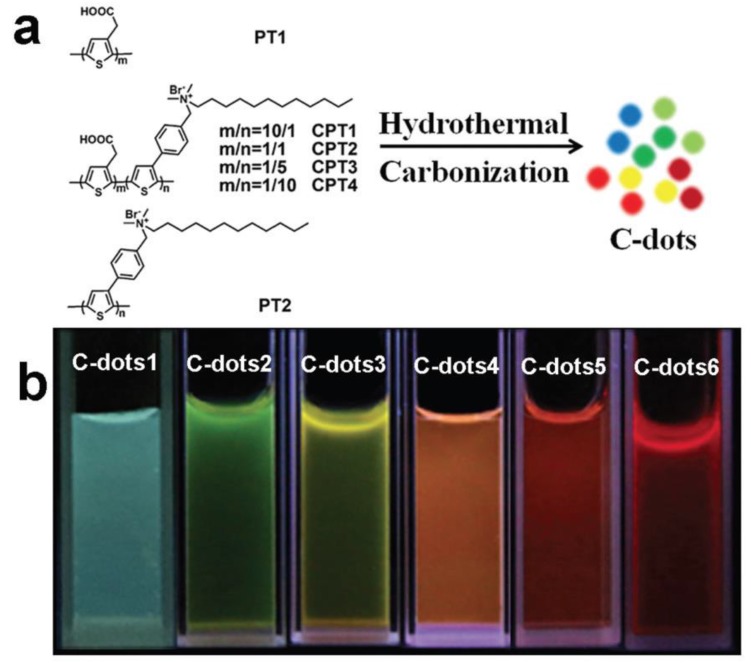
Tunable multicolour carbon dots with polythiophene: (**a**) Synthetic routes of C-dots; (**b**) emission colours of various C-dots under UV light. (Reproduced with permission from ref. [34]. Copyright 2016 The Royal Society of Chemistry, Cambridge, UK).

Polythiophene (PT) surely does not belong to the group of the most commonly used polymers for carbon dots. However, its derivatives have been successfully employed by Guo et al. to tune carbon dots to longer wavelengths [34]. Two different PT derivatives, first with carboxyl groups and second with quaternary ammonium salts, were used for producing co-doped carbon dots via a hydrothermal process. Co-doping was based on the presence of oxygen for the first case and nitrogen for the second along with sulphur on PT itself. Final dots exhibited tunable multicolour luminescence with emission wavelengths spanning across the visible spectrum up to the edges of the NIR region (Figure 5). Namely, six different materials were produced having PL peaks’ maxima at 482, 524, 570, 609, 628, and 680 nm, whereas the same excitation wavelength (400 nm) was applied to all.

#### 3.3.2. Near Infrared

**Figure 6:**
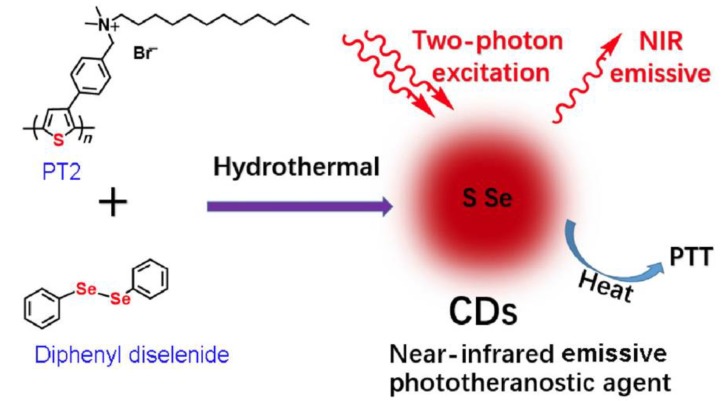
Schematic illustration for the preparation and working mechanism of two-photon-excited NIR-emissive S, Se-*co*-doped carbon dots by polythiophene and diphenyl diselenide. (Reproduced with permission from Reference [33]. Copyright 2017 Springer Nature, Basingstoke, UK).

PT has been also used by Lan and co-workers so as to induce sulphur doping [33]. A hydrothermal route was applied where PT was mixed with diphenyl diselenide to get S, Se-*co*-doped carbon dots (Figure 6). Interestingly, the obtained co-doped carbon dots exhibited a broad UV-Vis absorption ranging from 350 to 700 nm with a maximum centred at ~525 nm. Therefore, their photoluminescence rationally enters the NIR region. Whereas it is not discussed in detail, and in fact this behaviour cannot exactly be associated with either S or Se, it is quite intriguing that a simple hydrothermal process based on PT can lead to carbon dots with these optoelectronic properties. Moreover, the produced *co*-doped carbon dots were efficient for two-photon-excitation exhibiting in advance a photothermal conversion efficiency of ~58.2%, which is amongst the highest reported values for carbon nanostructures and comparable to that of Au nanostructures. Thus, these dots could be used for either NIR bioimaging or even photothermal therapy regardless their extremely low quantum yield of 0.2%. Furthermore, the few recent reports on the use of PT and its’ derivatives for tuning the fluorescence emission to lower energies reveal the potential of sulphur-bearing polymers in this topic.

**Figure 7:**
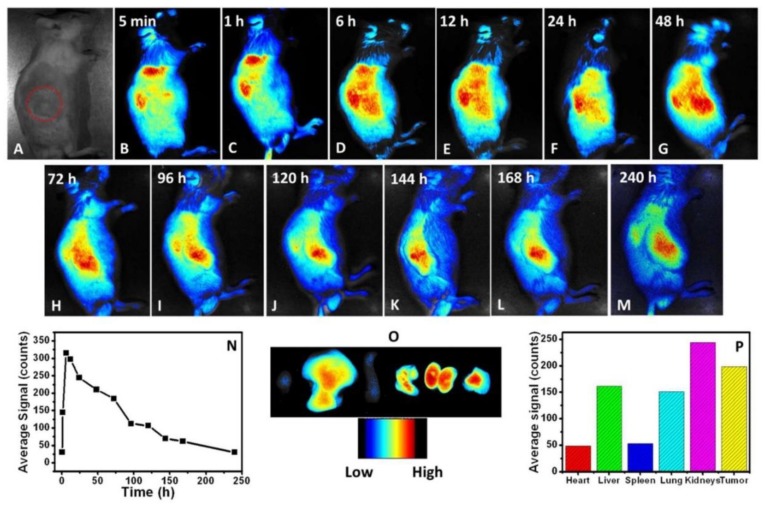
PEGylated carbon dots for NIR bioimaging: Time based in vivo red fluorescence images of BALB/c mouse bearing CT26 tumours after the intravenous (i.v.) injection of CyCD (the tumour is circled with a red dotted line). (**A**) 0 min (taken under natural light; the red circle marks the position of tumour); (**B**) 5 min; (**C**) 1 h; (**D**) 6 h; (**E**) 12 h; (**F**) 24 h; (**G**) 48 h; (**H**) 72 h; (**I**) 96 h; (**J**) 120 h; (**K**) 144 h; (**L**) 168 h; and (**M**) 240 h; (**N**) Average fluorescence intensity of the tumour area as a function of time; (**O**) Ex vivo imaging of the tissues 48 h post-injection (from left to right: heart, liver, spleen, lung, kidneys, and tumour); (**P**) Average fluorescent intensity of heart, liver, spleen, lung, kidneys, and tumour. (Reproduced with permission from Reference [25]. Copyright 2016 American Chemical Society).

Carbon dots, with better quantum yield reaching 5.7%, for analogous NIR and theranostic applications were prepared once again by a solvothermal method from PEG and a cyanine dye [2-((*E*)-2-((*E*)-2-chloro-3-((*E*)-2-(1-(2-hydroxyethyl)-3,3-dimethylindolin-2-ylidene)-ethylidene)cyclohex-1-en-1-yl)vinyl)-1-(2-hydroxyethyl)-3,3-dimethyl-3H-indol-1-ium iodide, CyOH] [25]. The hydrophobic cyanide dye, which in fact is a NIR chromophore, was encapsulated by PEG, hence not only a hydrophilic product was obtained but also its stability was enhanced. So, even though PEG’s contribution to the optoelectronic properties of the final carbon dots seems minimum according to the relevant presented UV-Vis and PL spectra, its significance is high because without PEG the cyanide dye cannot be used in such a way. In addition, carbon dots display high photothermal conversion efficiency (38.7%) along with preferential uptake at tumours, which settles them as ideal candidates for NIR bioimaging and photothermal therapy (Figure 7).

**Figure 8:**
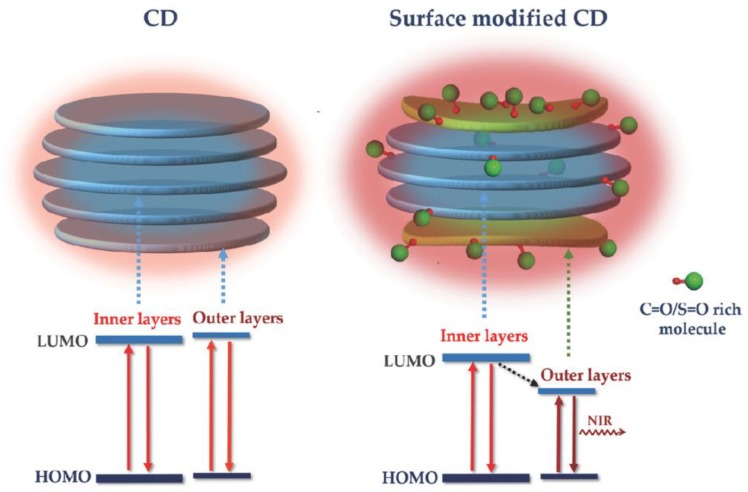
Schematic illustration of structure and energy level alignments of nontreated carbon dots (left column) and modified carbon dots with S=O/C=O-rich molecules/polymers (right column). The red (oxygen atom) and green double-bonded balls represent the C=O/S=O-rich molecule/polymer. (Reproduced with permission from Reference [38]. Copyright 2018 John Wiley & Sons).

Another recent fascinating report for carbon dots with PL emission at the NIR region is the one by Li et al. [38]. Polyvinylpyrrolidone (PVP) was used for the surface modification of carbon dots prepared by citric acid and urea under solvothermal conditions. Compared to the previously discussed studies for NIR [25,33], this one reports an enhanced quantum yield of 10% for in fact a direct excitation in the NIR-I window since the dots absorb effectively light above 700 nm. This is crucial for producing efficient probes for imaging in NIR-I window as achieved emissions for single-photon excitation are more intense. In addition, experiments have shown that under NIR excitation, PVP-modified carbon dots also exhibit enhanced NIR emission, with the intensity of this emission advancing with increasing PVP concentration. Furthermore, excitation at the second biological window (NIR-II window) by a 1400 nm femtosecond laser generates simultaneous two-photon-induced NIR fluorescence emission and three-photon-induced red fluorescence emission of carbon dots in dimethyl sulfoxide. Finally, the NIR absorption and enhanced fluorescence have been associated with the surface engineering of the carbon dots with molecules or polymers rich in sulfoxide/carbonyl groups, namely electron-acceptor groups, which influence the optical bandgap and promote electron transitions under NIR excitation (Figure 8). This wide opens a novel field in exploiting relevant groups-bearing polymers on carbon dots topic.

**Figure 9:**
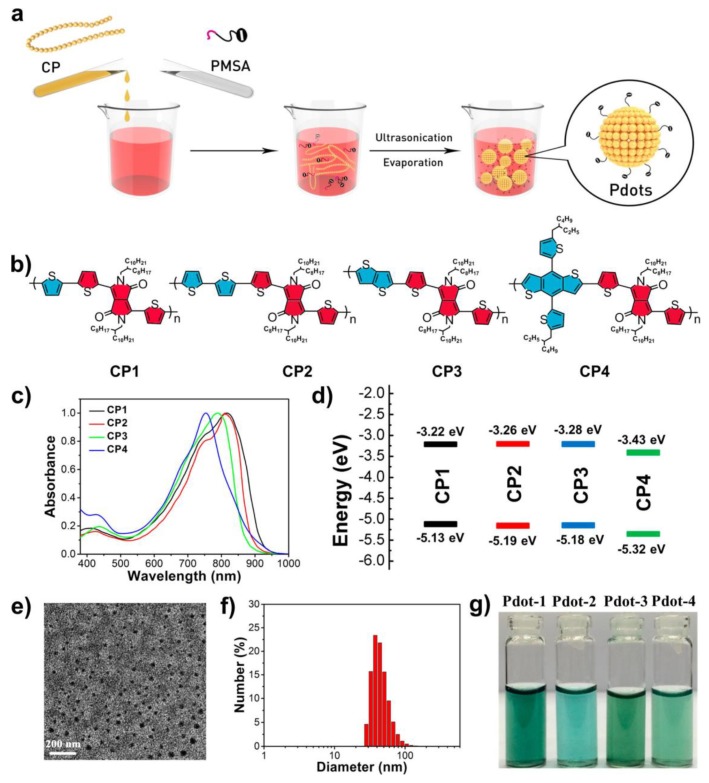
(**a**) Schematic illustration of preparation for Pdots; (**b**) Molecular structures of CP1-CP4; (**c**) UV-vis-NIR absorption spectra of CPs 1–4 in chloroform; (**d**) Band diagram representing the HOMO and LUMO levels of CP1-CP4 determined by cyclic voltammetry; (**e**) Representative TEM images of Pdot-1; (**f**) Size distribution histogram of Pdot-1 by DLS measurement; (**g**) Photographic images of Pdots 1–4 in aqueous solutions (25 µg/mL) after one month of storage at 4 °C. (Reproduced with permission from Reference [39]. Copyright 2016 American Chemical Society).

An even better photothermal conversion efficiency compared to the previous discussed was achieved by Li and co-workers [39]. Poly(styrene-*co*-maleic anhydride) was coprecipitated with varied diketopyrrolopyrrole-containing conjugated polymers bridged with different thiophene units (Figure 9). The resulting polymeric carbon dots exhibited broad absorption from 600 to 900 nm. However, and in contrast with the previous reports except Reference [33], these dots showed almost no fluorescence emission, indicating that the main pathway for excited-state deactivation is the non-radiative decay. Hence, the supressed energy loss during deactivation endows the dots with high efficiency to convert light (photon energy) to heat, and thus the photothermal conversion efficiency climbs to 65%. A similar explanation may apply as well for the efficiency found in Reference [33]. As a result, the formed polymeric carbon dots are excellent candidates for photothermal therapy. In this direction, their theranostic performance was examined in both in vitro and in vivo experiments. Carbon dots were irradiated with a NIR laser (808 nm) at a low power density (0.5 W/cm^2^) for 5 min and the tumour temperature raised to 53−68 °C, which is enough to kill the tumour cells. In fact, tumour was 100% eliminated with no observed recurrence, which highlights the potential of relevant materials for non-invasive cancer treatment.

### 3.4. Enhancing UV-A Absorption

**Figure 10:**
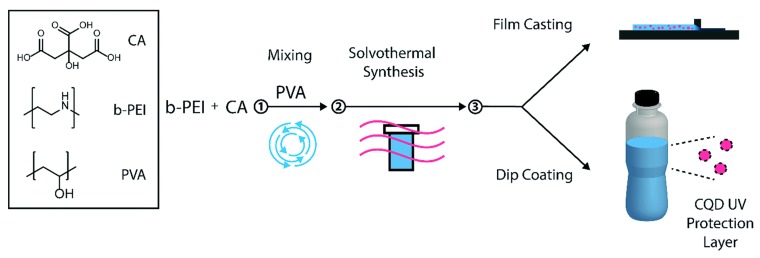
Process overview of CQD–PVA composite film synthesis. ① Citric acid (CA), branched polyethylene imine (b-PEI), and polyvinyl alcohol (PVA) mixed in water; ② Solvothermal synthesis; ③ Film casting by spiral bar coating and film drying. Alternatively, a glass vial was coated with the CQDs–PVA solution by dip coating. (Reproduced with permission from Reference [40]. Copyright 2017 The Royal Society of Chemistry).

Another very interesting employment of polymers in carbon dots is described by Hess et al. [40]. Using citric acid as carbon source, branched polyethyleneimine (b-PEI) as passivation agent, and PVA as a multitask precursor, they managed to develop carbon dots with enhanced UV-A absorption. It was deduced that both b-PEI and PVA were essential for the development of the efficient UV-absorbing carbon dots. In fact, it was shown that the level of the UV-absorption enhancement was related to the weight percentages of the polymers for concentrations up to 5 and 10 wt.% for b-PEI and PVA respectively. Further experiments revealed that PVA in the pristine synthesis solutions increased viscosity which was associated with the formation of isolated dots with distinct emission wavelengths. As a result, carbon dots with relatively narrow and intense absorbance bands on the UV-A region were obtained. Furthermore, carbon dots dispersed in PVA could effectively form films as well proper solutions for dip coating technique so as to create efficient UV-A blockers. The latter films and coatings demonstrated a pH-independent behavior and could block over 90% of the UV-A radiation while possessing high transparency in the visible range (>80%). Hence, these films could be used in transparent food packaging applications (Figure 10). Moreover, unexplored properties of these carbon dots may be studied in future works since the authors of Reference [40] did not deal at all with their fluorescence properties. Relevant UV-blocking films were produced by Konwar and colleagues by using chitosan as passivation and film-making agent [71]. However, in this case the transparency of the films was higher (about 20%), whereas significant part of the visible spectrum (up to 600 nm) was blocked too.

### 3.5. Narrowing Emission Bandwidths

**Figure 11:**
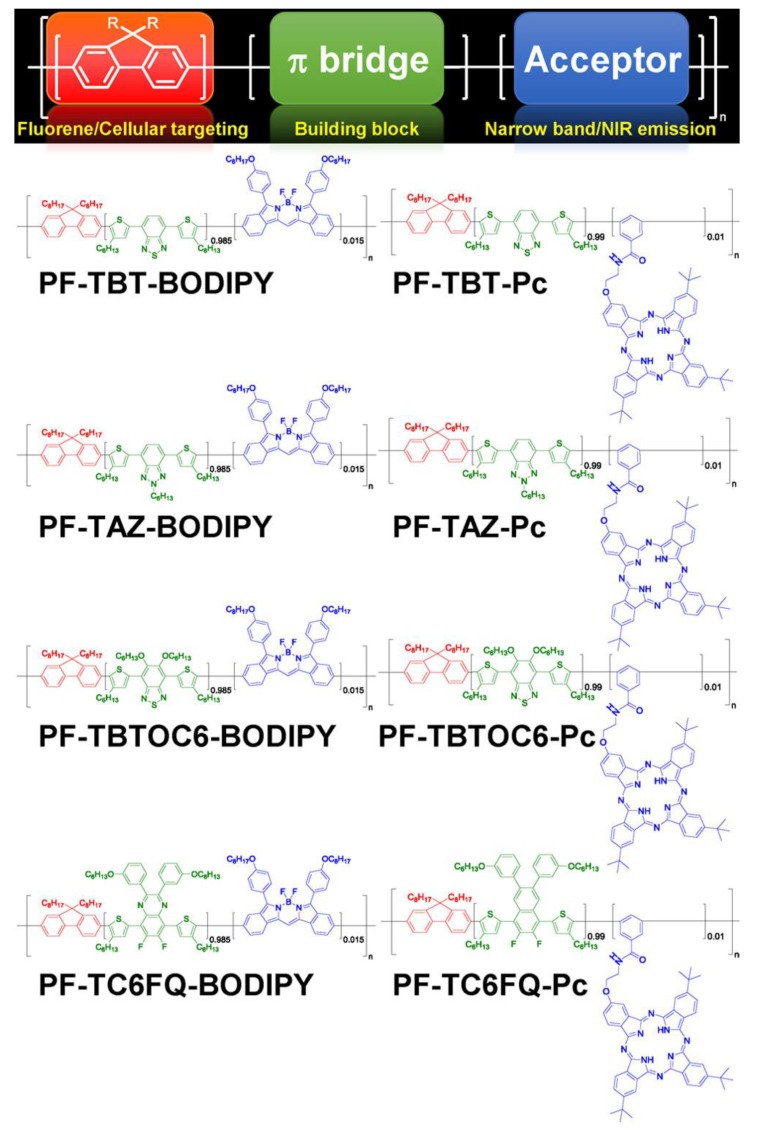
Chemical structures of a series of narrow-band NIR polymers used for producing polymer-based carbon dots. (Reproduced with permission from Reference [41]. Copyright 2017 American Chemical Society).

Typical emission spectra of carbon dots demonstrate broad bands and sometimes multiple peaks, or peaks accompanied by shoulders. The full-width at half-maximum (FWHM) of these bands is usually around 80 to 100 nm. This means that most of the times there is not a characteristic wavelength for specific dots where the latter emit light. As a result, in case of complex systems or mixtures of different emitters, distinction becomes impossible. Not to mention that for optoelectronic applications, carbon dots must have pretty good and discrete optical properties. To surpass this issue, the fluorophores must have as narrow as possible emission bandwidth. Hence, whereas several research groups have managed to obtain carbon dots with tuned luminescence at longer than blue wavelengths or even in the NIR region as described in the relevant subsection, a vital step further is to achieve narrow bandwidths for these emissions along with efficient quantum yield. In this direction, the work by Ke and colleagues offers a key input [41]. Their article describes the design and synthesis of donor−bridge−acceptor-based polymers for producing semiconducting carbon dots (Figure 11). As readily understood by just seeing Figure 11, their approach has a serious drawback for most researchers working on carbon dots, namely the use of organic−polymer chemistry to build these block *co*-polymers. Nevertheless, the presented results are impressive since following their method, they managed to obtain polymeric carbon dots with essential quantum yield in the NIR region up to 33%, while the FWHM of the fluorescence emission peaks could reach values as low as 29 nm. Both these values (33% and 29 nm) are benchmarks for NIR emissive carbon dots. Hence, the proposed molecular engineering approach for NIR emissive carbon dots seems to be highly effective and at least in this publication is based exclusively on polymer science. This may give an advantage to polymer scientists in producing advanced carbon dots in near future having also in mind that we are dealing with the crucial in vivo biological imaging in the NIR range. Regarding narrowing emission bandwidths, it is worth noting also that the purification of carbon dots by techniques as solid phase extraction, etc., leads to materials with advanced and more confined optical properties as narrow peaks [72].

## 4. Perspectives for Polymers in Carbon Dots

The aforementioned examples of how polymers contribute to the tuning of carbon dots’ optical properties, signify their importance on the progression achieved on the field. Carbon dots seem to have “indefinite” limitations with respect to their characteristics upon proper modifications: from ns-scale fluorescence to nearly s-scale phosphorescence; from low quantum yield values up to 94%; from blue emission to NIR emission; from low light absorption to forming cut-off blockers; from unclear emission peaks to narrow emission bandwidths. In addition, the advanced carbon dots may be employed in numerous applications. Many of these improvements, if not all, can be accomplished by engaging polymers. Thus, future research activities should focus on exploring polymers’ contribution.

Of high importance would be a systematic study where various polymers would be used for preparing or functionalizing carbon dots while keeping the rest affecting factors the same [73]. Moreover, in my view and based also on previous works as refs. [33,38], there are two specific polymer families that might endow carbon dots with elegant characteristics. Polymers with heteroatoms as S, P, B, or even further elements as Se, and benzene ring containing polymers. The first group of polymers may modify and alter carbon dots’ features in multiple ways, adding also doping on controlled manner as the incorporated heteroatoms will have a specific nearly standard environment which might lead to more precise properties, as for example narrow emission bandwidths [59,74], compared to doped carbon dots originating from inorganic precursors where the random nucleation can drive to numerous pathways and structures. Furthermore, heteroatom-containing polymers are expected to enhance the nonlinear optical properties of carbon dots [75], a topic not discussed in this review due to lack of relevant publications, with limited literature not dealing with polymer-based or functionalized carbon dots [76,77,78,79,80,81]. The second group of polymers might again lead to more defined structures due to steric hindrance whereas can introduce countless π electrons which are essential for the desired electronic transitions and observed optoelectronic phenomena of these materials. Finally, Reference [41] renders polymer science-based molecular engineering a very promising approach for carbon dots and thus should be explored.

## 5. Conclusions

This review summarizes the recent advancements on the use of polymers for carbon dots focusing on the manipulation and tailoring of their optoelectronic properties. Thus, it has been demonstrated that by employing various polymers as PEG, PVA, PEI, as well less common ones for the field of carbon dots as PT and PVP, the optical features of carbon dots can effectively be tuned. Tuning of optoelectronic properties includes expanding emission lifetimes for achieving phosphorescence, advancing quantum yields for brighter emissions, tuning absorption/emission wavelengths both in the visible and NIR regions for multiple applications, and mainly bioimaging and photothermal therapy for NIR, enhancing UV-A absorption for efficient UV-blockers and filters, and narrowing emission bandwidths for multiplexed biological detection and imaging. Given the versatility of carbon dots and the synthetic abundance of polymers, this topic is wide open to further improvements and developments. The bottom line of the review is that polymers may and should be fully exploited for obtaining tailor-made carbon dots.

## Figures and Tables

**Table 1:** List of polymers used in recent literature for carbon dots materials.

Polymers	Notes/Specific Applications	Ref.
PEG and PVA	Expanded emission lifetime-Phosphorescence-Data encryption [19]	[19,20]
PU ^1^	PU suppresses nonradiative transitions-Phosphorescence	[21]
PEG	Advancing quantum yield (QY) [22,23,24], Tuning emission [25,26]	[22,23,24,25,26]
Chitosan and PS ^2^	Advancing QY by attaching carbon dots on PS	[27]
PEI	Advancing QY [28,29,30], Tune emission across visible [31,32]	[28,29,30,31,32]
PT ^3^	Tune emission to visible and/or near-IR	[33,34,35,36]
PVP ^4^	Advancing QY [37], Tune emission to near-IR [38]	[37,38]
PSMA ^5^	Tune absorption to near-IR-Photothermal therapy	[39]
b-PEI and PVA	Enhancing UV-A absorption	[40]
Various	Narrowing emission bandwidth	[41]

^1^ Polyurethane; ^2^ Polystyrene; ^3^ Polythiophene; ^4^ Polyvinylpyrrolidone; ^5^ Poly(styrene-*co*-maleic anhydride).
